# Detection of Contralateral Malignancies on Breast MRI

**DOI:** 10.7759/cureus.66510

**Published:** 2024-08-09

**Authors:** Alan Y Xu, Mariam Hanna

**Affiliations:** 1 Radiology, University of Florida College of Medicine, Gainesville, USA

**Keywords:** breast cancer, contralateral, bilateral, synchronous, mammography, mri, breast

## Abstract

Introduction: Women with unilateral breast cancer are at increased risk for having simultaneous cancer of the contralateral breast. Overall, earlier detection of contralateral breast cancer prevents the burden of additional surgery or chemotherapy rounds and is associated with higher overall survival. However, MRI screening for the contralateral breast is seldom done following an initial unilateral breast cancer diagnosis. The purpose of this study is to retrospectively evaluate patients with known, biopsy-proven malignancy who went on to obtain a breast MRI and were later found to have cancer of the contralateral breast.

Methods: This was a retrospective study that reviewed the charts of women aged over 18 years who were determined to have synchronous bilateral breast cancer from January 2017 to January 2022 at the University of Florida, Gainesville, FL. The study extracted data from this institution’s cancer registry database, which provided information on patients with breast cancer diagnoses. The study conducted a review of mammography (MAM) and MRI imaging reports to ascertain the presence or absence of contralateral breast cancer identified by each respective imaging modality. Surgical pathology reports from the biopsy of the contralateral breast were reviewed to obtain information on the histological type of cancer and TNM (tumor, node, metastasis) staging.

Results: Of the 17 cases in which MAM missed contralateral cancer, follow-up MRI detected contralateral malignancy in 12 cases (70.59%) and subsequently changed management, resulting in additional imaging, biopsy, and eventual diagnosis and treatment of contralateral breast cancer. Examining the number of contralateral breast cancers detected by patients who had undergone MAM followed by MRI and those who had only undergone MAM, the study found that the detection rate of contralateral breast cancer from MAM was 45.45% (15/33). The tumor stages of the missed cancers were all T1 or Tis stage with one T1mi, and there was no nodal involvement.

Conclusion: In addition to its utility in staging breast cancers, MRI also has the superior ability to detect otherwise undetected contralateral breast malignancy. This retrospective study found that MRI imaging led to a considerable increase in the detection of contralateral cancer. The study found that these undetected contralateral breast cancers by MAM were often of lower staging with no nodal involvement, highlighting the opportunity for MRI to assist in early cancer detection while the patient's prognosis is still good. Its high cost should be balanced with staging and occult malignancy detection utility in future practice.

## Introduction

A diagnosis of cancer in both breasts within six months is known as bilateral synchronous breast cancer, and it accounts for 0.2%-2% of all breast cancers [1]. Women with unilateral breast cancer are at increased risk for having simultaneous cancer of the contralateral breast [2]. It is estimated that up to 10% of women undergoing treatment for unilateral breast cancer had contralateral breast cancer despite normal findings on clinical and mammographic (MAM) examination [2]. Prospective studies in the literature have shown variable MRI detection rates for otherwise undetected contralateral breast cancers ranging from 3% to 24% [2,3]. Overall, earlier detection of contralateral breast cancer prevents the burden of additional surgery or chemotherapy rounds and is associated with higher overall survival [2,4]. However, MRI screening for the contralateral breast is seldom done following an initial unilateral breast cancer diagnosis.

The purpose of this study is to retrospectively evaluate patients with known, biopsy-proven malignancy who went on to obtain a breast MRI and were later found to have cancer of the contralateral breast. Analysis of these data will help determine the MAM detection rate of contralateral malignancy as well as MRI. In addition, it will allow us to examine patterns and characteristics of breast cancers that are undetected by mammography. Overall, this study will aid criteria for the utilization of breast MRI following the diagnosis of unilateral breast cancer.

## Materials and methods

This was a retrospective study that reviewed charts of women aged over 18 who were determined to have synchronous bilateral breast cancer from January 2017 to January 2022 at the University of Florida, Gainesville, FL. The current study extracted data from this institution’s cancer registry database, which provided information on patients with breast cancer diagnoses. The diagnosis of bilateral malignancy was determined by identifying patients with Breast Imaging-Reporting and Data System 6 (BI-RADS-6) scores in both breasts. For classification as synchronous, the diagnosis of malignancy for each breast had to occur within a six-month timeframe of each other.

The current study conducted a review of MAM and MRI imaging reports to ascertain the presence or absence of contralateral breast cancer identified by each respective imaging modality. A positive finding was defined as the presence of a suspicious lesion documented in the contralateral breast in the imaging report, while a negative finding indicated the absence of any such suspicious lesion, characterized as benign. Mammography imaging reports were also used to obtain information on the breast density of each patient. Surgical pathology reports from the biopsy of the contralateral breast were reviewed to obtain information on the histological type of cancer and TNM (tumor, node, metastasis) staging system.

Statistical analysis was conducted with the use of SAS JMP software (JMP Statistical Discovery LLC, Cary, NC). Descriptive statistics were used to summarize the characteristics of contralateral breast cancer. The odds ratio (OR) was calculated to assess the association between patient and cancer characteristics with the probability of detection by MRI and MAM. A two-tailed p-value <0.05 was considered statistically significant.

## Results

**Figure 1:**
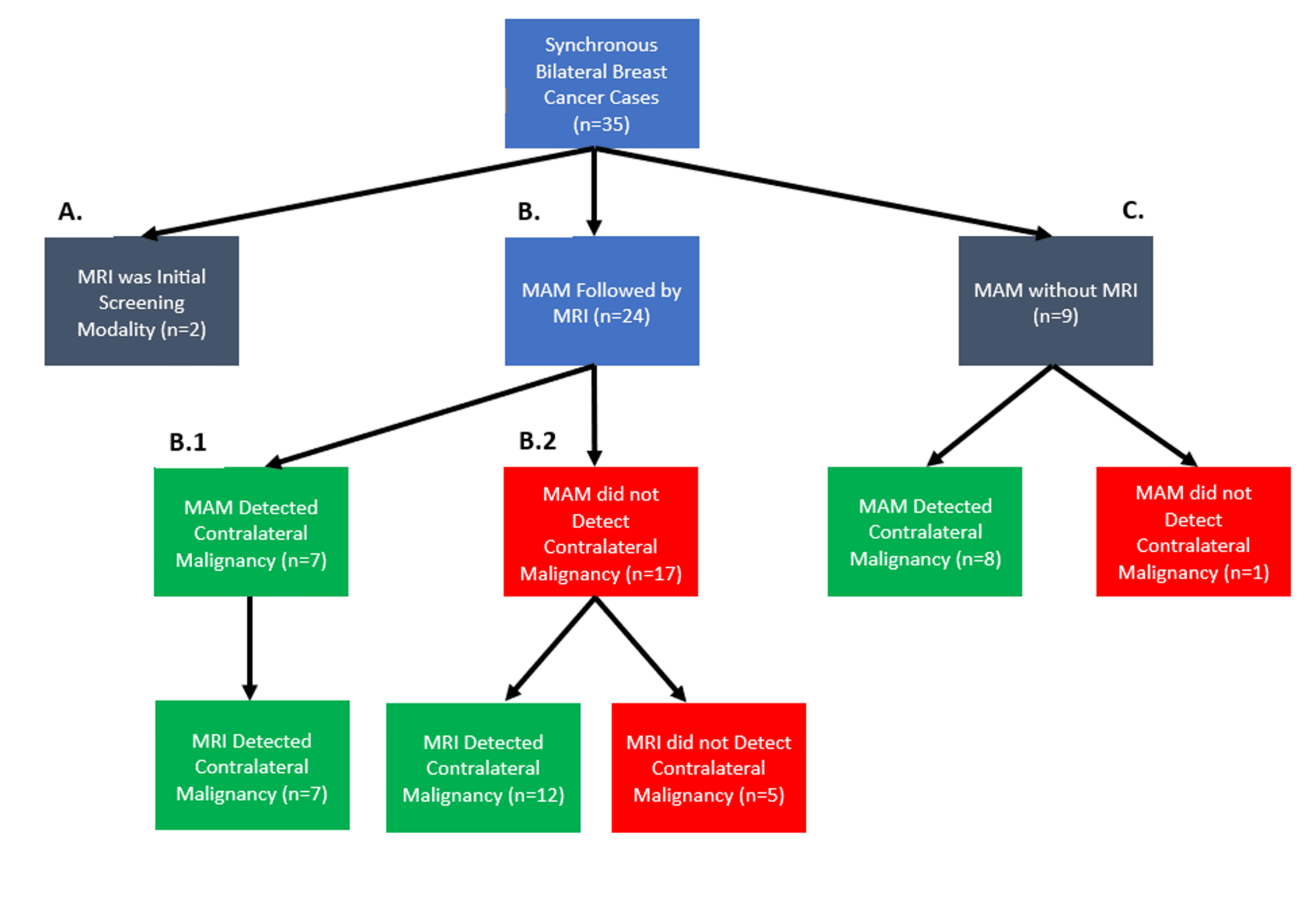
Flowchart of imaging screening modalities for bilateral breast cancer and the detection rate of contralateral breast cancer MAM: mammography

Thirty-five patients had synchronous bilateral breast cancer. In two cases, MRI was the initial screening modality, which detected bilateral breast cancer (Figure 1A). In nine cases, MAM was the initial screening modality, but follow-up MRI staging was not performed (Figure 1C). In eight of these nine cases (89%), initial MAM detected contralateral breast cancer (Figure 1C). In one case, MAM did not detect contralateral breast cancer, but it was later found after sampling tissue from a bilateral mastectomy (Figure 1C). Twenty-four patients were initially screened with MAM and followed up with bilateral MRI for further staging (Figure 1B). Initial MAM screening detected contralateral breast cancer in seven of these 24 cases (29.17%), and subsequent MRI was concordant with this finding (Figure 1B.1). Of the 17 cases in which MAM missed contralateral cancer, follow-up MRI detected contralateral malignancy in 12 cases (70.59%) and subsequently changed management, resulting in additional imaging, biopsy, and eventual diagnosis and treatment of contralateral breast cancer (Figure 1B.2). In the remaining five out of 17 (29.41%) cases where MRI also did not detect contralateral breast cancer, the cancer was incidentally found after tissue samples from a prophylactic bilateral mastectomy were analyzed (Figure 1B.2). Examining the number of contralateral breast cancers detected by patients who had undergone MAM followed by MRI and those who had only undergone MAM, the study found that the detection rate of contralateral breast cancer from MAM was 45.45% (15/33).

**Figure 2:**
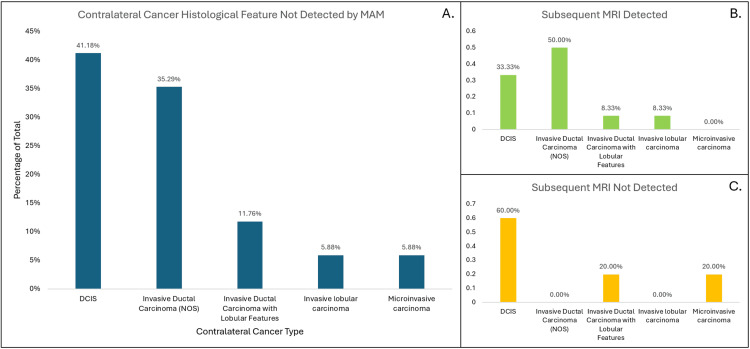
Bar plot of the proportion of types of cancers missed by MAM/US of the contralateral breast MAM: mammography; DCIS: ductal carcinoma in situ

The study analyzed the pathology reports of excised breast tissues in the 17 cases where MAM/US was performed (but missed the contralateral diagnosis) with subsequent MRI imaging. The histological features of the contralateral cancers that were not detected by mammography are shown in Figure 2. The most prevalent types were ductal carcinoma in situ (DCIS) and invasive ductal carcinoma not otherwise specified (NOS) at 41.18% and 35.29%, respectively.

The study examined the TNM staging for contralateral breast cancer in the pathology reports. The results are shown in Table 1. All tumor stages were T1 or Tis, except for one patient who had microinvasive carcinoma (T1mi). For all patients with contralateral invasive carcinoma, there was no nodal involvement, and thus nodal staging was N0.

**Table 1:** Contralateral cancer MAM/MRI detection rate versus patient age and tumor staging Percentages are reported as column percentages. Category B (scattered fibroglandular densities) was considered non-dense, and Categories C (heterogenous dense) and D (extremely dense) were grouped as dense. MAM: mammography

	Detected by MAM	Not detected by MAM	Not detected by MAM + detected by MRI	Not detected by MAM + not detected by MRI
Average age (years) (Mean±SD)	65.7 ± 15.6	59.6 ± 11.3	62.9 ± 9.9	51.8 ± 11.4
Tis (N, %)	-	7 (41.2)	4 (33.3)	3 (60.0)
T1a (N, %)	-	4 (23.5)	3 (25.0)	1 (20.0)
T1b (N, %)	-	3 (17.6)	3 (25.0)	0 (0.0)
T1c (N, %)	-	2 (11.8)	2 (16.7)	0 (0.0)
T1mi (N, %)	-	1 (5.9)	0 (0.0)	1 (20.0)

The study analyzed the likelihood of detection by mammography and subsequent MRI based on cancer type (in situ carcinoma versus invasive carcinoma) as well as breast density as determined by initial mammography (dense versus non-dense breast tissue). The values are shown in Table 2. There was no statistically significant difference between cancer type and breast density and likelihood of detection by mammography or MRI.

**Table 2:** Detection by MAM/MRI by carcinoma type and breast density Microinvasive carcinoma was included in the invasive carcinoma category. Percentages are reported as column percentages. Category B (scattered fibroglandular densities) was considered non-dense, and Categories C (heterogeneous dense) and D (extremely dense) were grouped as dense. P <0.05 was considered significant. MAM: mammography; OR: odds ratio

	Detected by MAM (N,%)	Not detected by MAM (N,%)	OR (95% CI)	Not detected by MAM + detected by MRI (N,%)	Not detected by MAM + not detected by MRI (N,%)	OR (95% CI)
Invasive carcinoma	-	10 (58.8)	-	8 (66.7)	2 (40.0)	3 (0.35, 25.87)
In situ carcinoma	-	7 (41.2)		4 (33.3)	3 (60.0)	
Dense breast tissue	10 (66.7)	10 (58.8)	1.4 (0.33, 5.93)	7 (58.3)	3 (60.0)	0.93 (0.11, 7.82)
Non-dense breast tissue	5 (33.3)	7 (41.2)		5 (41.7)	2 (40.0)	

## Discussion

While it is currently standard practice to evaluate the contralateral breast in women with unilateral breast cancer with clinical breast examination and mammography, it is not standard to follow up with bilateral MRI imaging of both breasts. This retrospective study found that mammography detected 45.5% (15/33) contralateral breast cancers. Subsequent MRI detected 70.59% (12/17) of the missed contralateral cancers.

The MAM-undetected contralateral breast cancers were predominantly DCIS and invasive ductal carcinoma NOS subtypes. Although dense breast tissue typically hampers the detection of breast cancers, the current study results indicate that breast density did not significantly influence whether contralateral breast cancer was detected via mammography [5]. The distinction between in situ and invasive carcinoma similarly had no impact on its detection. All tumor stages of contralateral breast cancer were T1 or Tis (ductal carcinoma in situ), with one patient having T1mi (microinvasive carcinoma). There was no nodal involvement in any of the cases. Although DCIS is non-invasive, it is likely to become invasive if untreated [6-8]. It is estimated that 25%-60% of untreated DCIS progresses to invasive ductal carcinoma [9]. The current study found that undetected contralateral breast cancers by mammography often present with lower staging and haven't spread to lymph nodes, a significant prognostic indicator for breast cancer. This underscores the importance of MRI in early cancer detection, thereby optimizing patient prognosis. Early identification of synchronous contralateral breast cancer can also mitigate the necessity for multiple treatments on different occasions, ultimately alleviating patient burden and reducing costs.

In the literature, two studies indicate sensitivities of 91% and 80%, along with additional diagnostic yields of 3.1% and 1%, respectively, for MRI in detecting breast cancer [2,10]. While the limitation of breast MRI is its lower specificity, some studies have found it to have a specificity of 88%, a false positive rate of 10.9%, and a very high negative predictive value of 99% [1]. Though some women may opt for contralateral mastectomy, negative findings of cancer in the contralateral breast on MRI and mammography may reduce the number of unnecessary mastectomies [1]. A study conducted on women with unilateral breast cancer found that the rates of contralateral prophylactic mastectomy increased from 3.4% to 6.8% from 2016 to 2019 [11]. Furthermore, MRI is considered superior to MAM and ultrasound in defining the extent of disease for staging, assessing nipple-to-tumor distance to decide on nipple-sparing mastectomy, and both nonsurgical and surgical planning of DCIS [12]. While the high cost of MRI remains a barrier to its widespread adoption and use as a first-line screening tool for breast cancer, its utility in staging and detecting occult cancers of the contralateral breast should not be overlooked.

Limitations of this study include the relatively small sample size and being a single-institution study. The prevalence of synchronous bilateral breast cancer is low, which yields a small sample to study, and we attempted to mitigate this by studying patients over the span of five years at our institution. All cases of MAM-missed contralateral breast cancer were found because MRI had detected them or because they were incidentally found on a contralateral prophylactic mastectomy. There is the possibility there are other cases of contralateral cancers that have yet to be detected if bilateral mastectomy was not pursued for these patients, which would have led to an overestimation of the detection rate of both MAM and MRI. 

## Conclusions

In addition to its utility in staging breast cancers, MRI also has a superior ability to detect otherwise undetected contralateral breast malignancies. This retrospective study found that MRI imaging led to a considerable increase in the detection of contralateral cancer. The current study found that these undetected contralateral breast cancers by MAM were often of lower staging with no nodal involvement, highlighting the opportunity for MRI to assist in early cancer detection while the patient's prognosis is still good. Its high cost should be balanced with staging and occult malignancy detection utility in future practice.
